# Frequent fragility of randomized controlled trials for HCC treatment

**DOI:** 10.1186/s12885-021-08133-8

**Published:** 2021-04-09

**Authors:** Hao Zhang, Jingtao Li, Wenting Zeng

**Affiliations:** 1 Department of Infectious Diseases, The Key Discipline of Gguangdong Province, The First Affiliated Hospital of Guangzhou Medical University, Guangzhou Medical University, #151 Yanjiang Road, Guangzhou, 510120 Guangdong Province China; 2 Department of liver diseases (I), The Hospital Affiliated to Shaanxi University of Chinese Medicine, Xianyang, 712000 Shaanxi Province China

**Keywords:** Fragility index, Randomized controlled trials, Endpoint

## Abstract

**Background:**

The fragility index (FI) of trial results can provide a measure of confidence in the positive effects reported in randomized controlled trials (RCTs). The aim of this study was to calculate the FI of RCTs supporting HCC treatments.

**Methods:**

A methodological systematic review of RCTs in HCC treatments was conducted. Two-arm studies with randomized and positive results for a time-to-event outcome were eligible for the FI calculation.

**Results:**

A total of 6 trails were included in this analysis. The median FI was 0.5 (IQR 0–10). FI was ≤7 in 4 (66.7%) of 6 trials; in those trials the fragility quotient was ≤1%.

**Conclusion:**

Many phase 3 RCTs supporting HCC treatments have a low FI, which challenges the confidence in concluding the superiority of these drugs over control treatments.

**Supplementary Information:**

The online version contains supplementary material available at 10.1186/s12885-021-08133-8.

## Background

Modern medicine is built on evidence-based clinical practice, with randomized controlled trials (RCTs) forming the foundation of such evidence. Because RCTs play important roles in governing clinical practice, the robustness of their results is critical. The results of clinical trials must be valid, reproducible, and repeatable; however, in the context of clinical research, reproducibility and replicability are generally under-researched topics. Historically, *P* values have been used to indicate statistical the significance of results in clinical trials [1]. Nevertheless, this approach has some significant limitations and has been heavily criticized for being simplistic, with frequent misapplication and misinterpretation [2].

The fragility index (FI) is a novel tool, which was developed to assess the robustness of statistically significant dichotomous outcomes from RCTs [3]. It is defined as the minimum number of patients receiving experimental treatment whose status would have to change from a non-event to an event to nullify a meaningful result. A higher FI represents a relativiely robust outcome and indicates that the statistical significance of a given outcome hinges on a greater number of events, whereas a lower FI indicates that the statistical significance of a given outcome depends on only a few events, which suggests a more fragile outcome.

The recommendation of new drugs or treatments for use in clinical practice, mainly depends on the results of phase 3 clinical trials. Thus, this study was performedto analysis to assess the wider implications of the FI in the findings of HCC treatments in phase 3 clinical trials.

## Methods

This study conducted a methodological systematic review of phase 3 RCTs for HCC treatment. The search terms used were (hepatocellular carcinoma OR hepatocarcinoma OR “liver cancer” OR HCC) AND (“phase 3” OR “phase III”). Only articles published in English were searched for using PubMed search engine and Medline database until August 1, 2019.

**Fig. 1:**
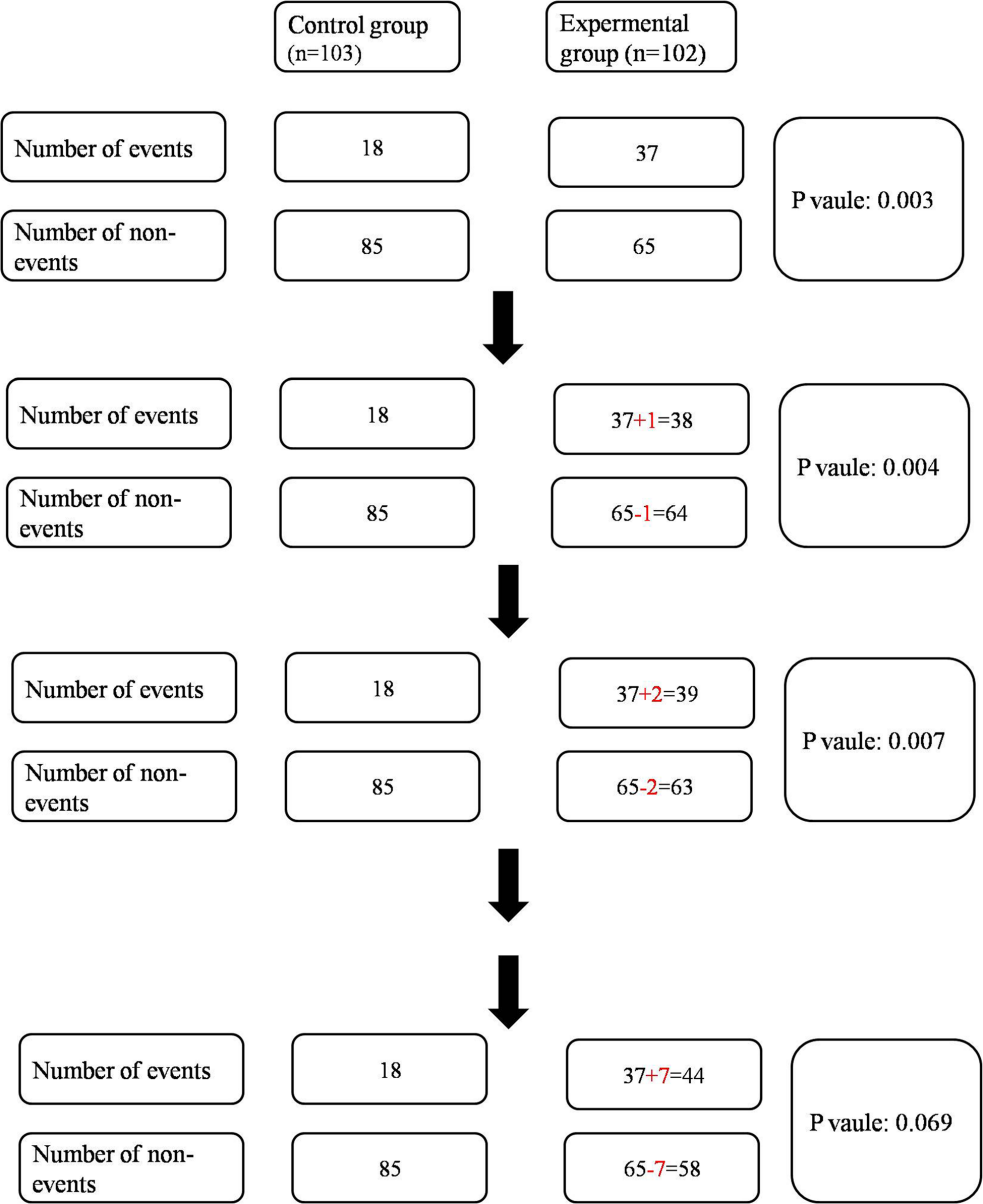
Example of fragility index calculation for the phase 3 trial SILIUS reported by Kudo M, et al [4]

For the FI analysis, only two-arm studies with randomization that reported significant positive results with primary or secondary outcomes were included. Data was obtained on trial design, trial number, and the observed numbers of events for the control and experimental groups in primary or secondary time-to-event outcomes. The FI was calculated from a two-by-two contingency table by the iterative addition of an event to the experimental group, which was determined using a web-based fragility calculator (available at http://www.clincalc.com/Stats/FragilityIndex.aspx). *P* values were calculated using Fisher’s Exact Test. A sample of FI is presented in Fig. 1.

The fragility quotient (FQ) is a metric, that accounts for the FI in the context of sample size [5]. It is described as the FI divided by the total sample size. The usefulness of the FQ lies in its ability to allocate an objective value to the results of subjective importance, and it may be assigned to an outcome with a given FI in a certain sample size [5]. In other words, the FQ assesses the robustness of the FI.

## Results

**Fig. 2:**
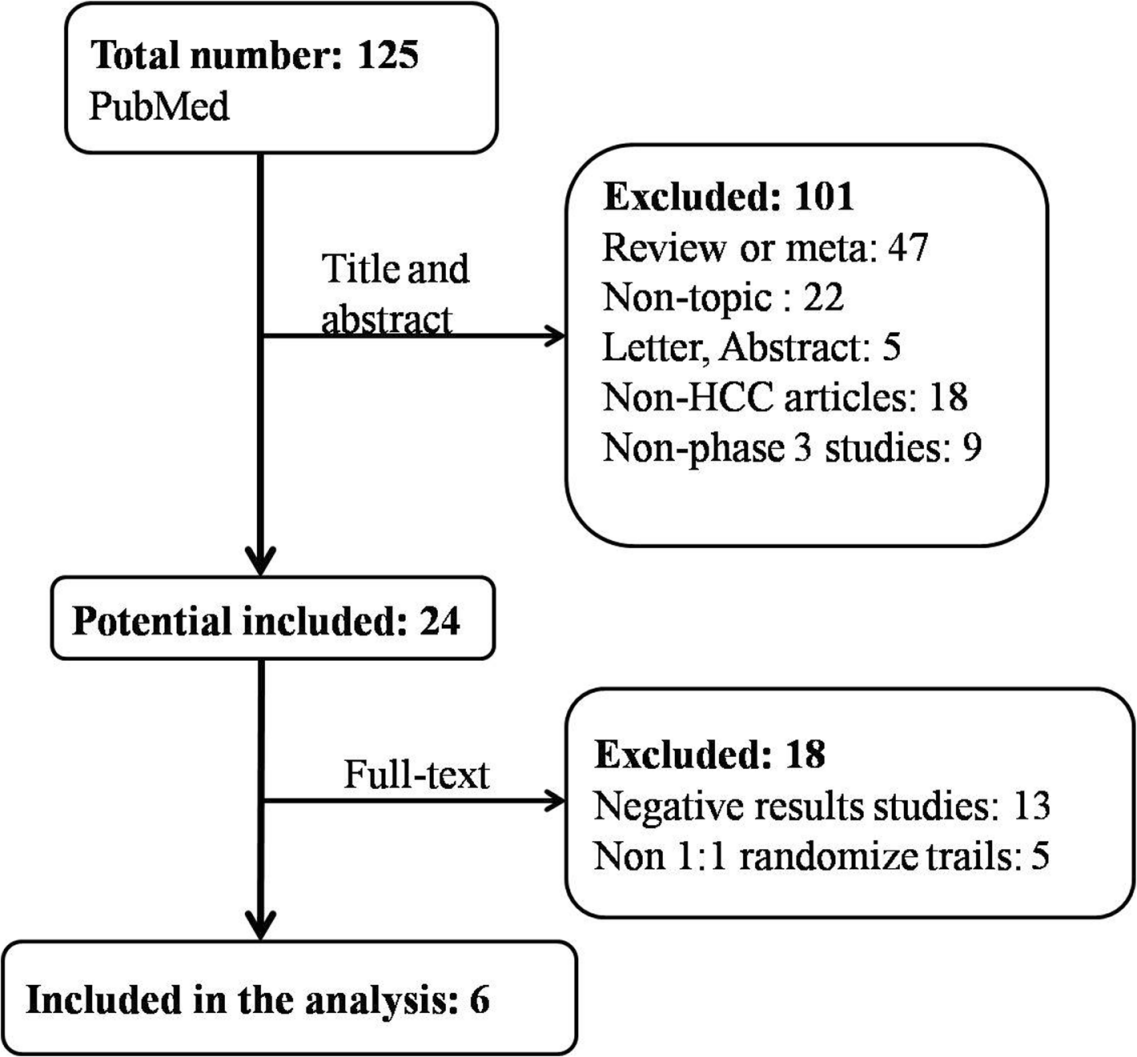
Flow chart for included studies

**Fig. 3:**
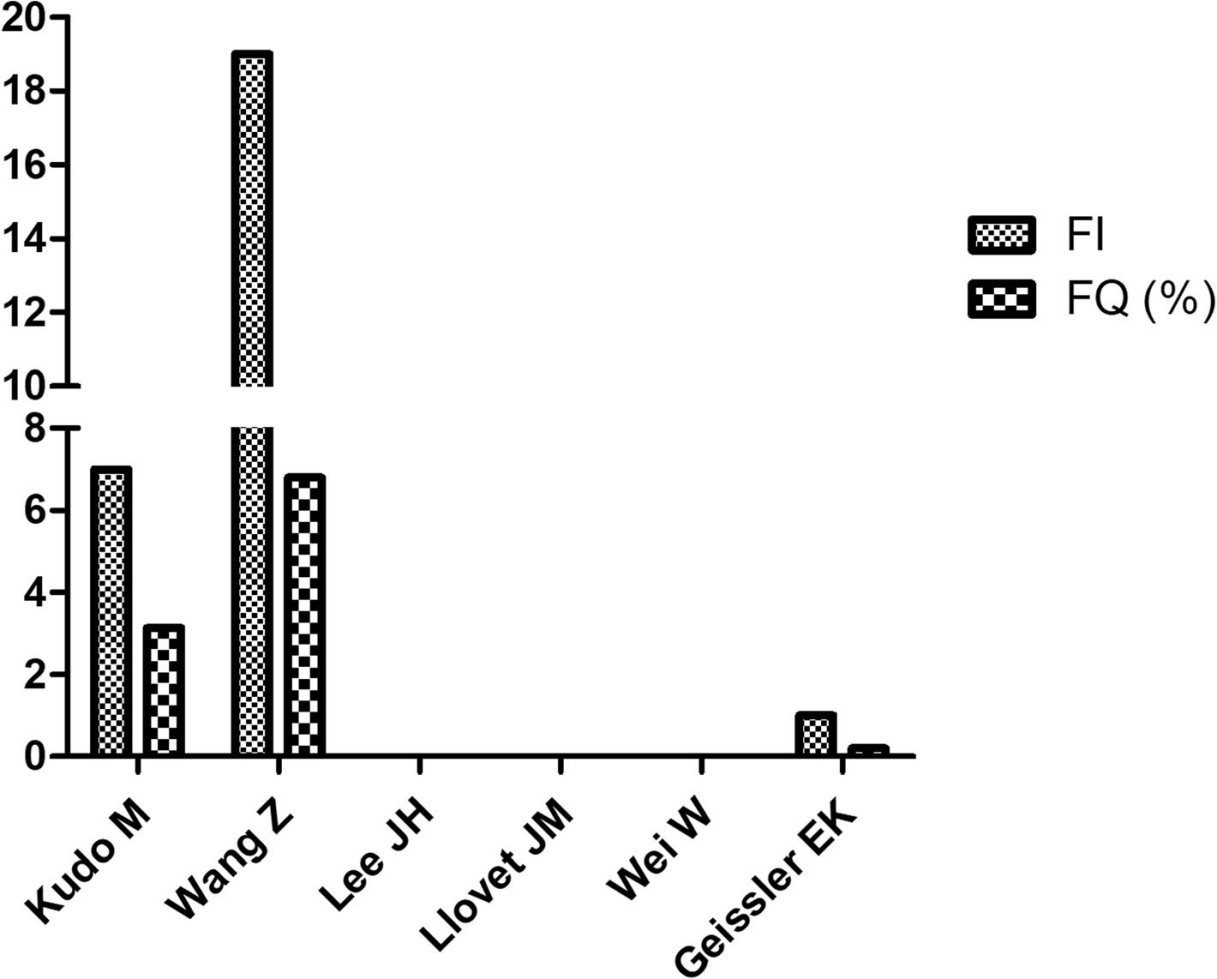
FI and FQ in the included studies

This study identified 125 records through a series of PubMed searches (Fig. 2). After an initial screening of abstracts and a full-text review of the studies, 6 articles were included in the fragility analysis (Table 1, Fig. 3) [4, 6–10]. The other five RCTs were excluded, as FI can only be calculated in RCTs that allocate 1:1 randomization (Supplementary Table 1). The median sample size for the 6 eligible RCTs was 257 (IQR 220.75–539), and the median FI for the 6 studies was 0.5 (IQR 0–10). The FI ≤ was 7 in 4 (72.73%) of 6 trials [7–10], and those trials had FQ < 1%.

**Table 1:** Fragility index calculated for 6 phase 3 trials with 1:1 randomization for HCC treatment

Author	Study name	Clinical Trial	Experimental Treatment vs. Control	Endpoint	Experimental sample size	Experimental event number	Control sample size	Control event number	P vaule	Fragility index	Fragility quotient
Kudo M et al. [4].	SILIUS	NCT01214343	Sorafenib plus HAIC (hepatic arterial infusion chemotherapy) vs. Sorafenib	Primary outcome: Overall response	102	37	103	18	0.003	7	3.41%
Wang Z et al. [6].	NA	NCT01966133	adjuvant TACE vs. No adjuvant TACE	Primary endpoint: Recurrence-free survival	140	46	140	82	0.01	19	6.79%
Lee JH et al. [7].	NA	NCT00699816	CIK cell agent vs. No CIK cell agent	Primary end point: Recurrence-free survival	114	69	112	59	0.01	0	0%
Llovet JM et al. [8].	SHARP	NCT00105443	Sorafenib vs. Placebo	Primary endpoint: Overall survival	299	44	303	33	0.00583	0	0%
Wei W et al. [9].	NA	NCT02788526	Hepatectomy plus TACE vs. Hepatectomy	Primary endpoint: Disease-free survival	116	83	118	85	0.02	0	0%
Geissler EK et al. [10].	NA	NCT0035586.	Liver transplantation with sirolimus vs. Liver transplantation	Secondary endpoint: Overall survival	252	242	256	234		1	0.20%

Five studies in the fragility analysis were for primary outcome results. Three (60%) had primary outcome trials with a FI of 0 (Fisher’s exact test *p* > 0.05), for which a stratified log-rank test was used to calculate the reported significant *P* value [7–9], and these three (60%) trials had an FQ < 1% [7–9]. The article with the highest FI fragility index of 19 was published in the *Clinical Cancer Research* [6]. However, this study was not a multiple center trial. The remaining 1 study was evaluated with inferior outcome results, whereas non significant differences were found in the primary outcome results. The study of the FI was 1, and the FQ was less than 1% [10].

## Discussion

To the best of our knowledge, FI investigation for HCC trials has not been performed. The FI has been evaluated in other RCTs, such as emergency medicine [11], giant cell arteritis, Clinical Practice Guidelines [12], and cardiac surgery field [13]. These studies consistently show that many RCTs are fragile, and several researchers have recommended that FI should be adopted in reporting clinical trial outcomes [12, 14], our study showed that most results from the randomized trials were far more fragile.

This analysis demonstrated that over 60% of the phase 3 trials supporting HCC treatments had a low FI; however, they are vulnerable to losing their significance with just a small change in the designation of a small number of events, often equating to < 1% of the sample size in an experimental group. As clinical practices or the use of drugs approved by Food and Drug Administration are developed on the results of phase 3 clinical trials, the change in the number of events required for fragility raises concerns about a statistical change in the results.

RCTs, particularly phase 3 clinical trials, are likely to remain an important evidence base for clinicians’ practice. Despite this, the statistical methodology used to establish significance in such clinical trials has barely evolved. In principle, the *P* value is an indication of the compatibility among data from a trial; a smaller P value implies a greater statistical incompatibility of the result with the null hypothesis (an estimation of no difference between the experimental and control group [15]). However, this approach has been greatly criticized for being simplistic, and has frequently been misinterpreted [16]. The log-rank test used in survival data analysis has advantage in that it accounts for events, but it relies on the assumption that the hazard ratio of two treatments remains constant over time. Fisher’s exact test, which is used to calculate the FI, has the disadvantage of not accounting for the time-to-event [17]. Thus, the FI is simplistic in its application and resolves some of these shortcomings.

Although the FI and FQ do provide a relative wealth of information when consider alongside other metrics, this study again emphasizes the limitations of the FI itself. First, clinical trials must obtain significant in effects in the treatment group, which means that treatment group got better results compared with control group. These trails could be included to be analyzed by the FI. Many non-inferiority studies cannot be included in this analysis, such as the E7080 trials of lenvatinib for HCC, which produced the same treatment results as sorafenib^22^. Second, because the FI relies on *P* value, it is essentially an extension of the most frequent approach to data analysis. Thus, it cannot be applied to an outcome of a continuous variable. Third, although many time-to-event outcomes are usually dichotomous, such as mortality, and survival, etc., the FI does not account for the difference in outcomes over time. Particularly in longer studies with variable follow-up time periods, analyses that account for time (such as a Kaplan–Meier curve, or a Cox proportional hazards model) are more appropriate than a simple binary outcome analysis. Fourth, our study shows a tendency of the inverse correlation between the FI and *p*-value, which is similar with previous FI studies [18, 19]. This might be the RCT studies included small number patients. Also, The FI was much higher as the samples increasing [20, 21]. Finally, there is no specific cut-off value or lower limit of the FI to classify a study as “either fragile” or “robust”.

## Conclusion

The outcomes of many phase 3, RCTs supporting HCC treatments with a low FI challenges the confidence in concluding the superiority of these drugs over control treatments.

## Supplementary Information

**Additional file 1: Table S1.** The exclusion cause and names of the excluded RCTs as FI can only be calculated in RCTs that allocate 1:1 randomization. Supplementary References.

## Data Availability

Data sharing is not applicable to this article as no datasets were generated or analysed during the current study.

## Abbreviations

HCC: Hepatocarcinoma
RCTs: Randomized controlled trials
FI: Fragility index
FQ: Fragility quotient
