# Supplementary material for: Frequent fragility of randomized controlled trials for HCC treatment
Source: BMC Cancer. 2021 Apr 9;21:389. doi: 10.1186/s12885-021-08133-8 (PMC8034173; doi:10.1186/s12885-021-08133-8)
Supplement: Supplementary file 1 — Additional file 1: Table S1. The exclusion cause and names of the excluded RCTs as FI can only be calculated in RCTs that allocate 1:1 randomization. Supplementary References. [file 12885_2021_8133_MOESM1_ESM.docx]

Supplementary table 1: The exclusion cause and names of the excluded RCTs as FI can only be calculated in RCTs that allocate 1:1 randomization

| Author | Study name | Clinical Trial | Experimental Treatment vs. Control | Endpoint | Experimental sample size | Experimental event number | Control sample size | Control event number |
| --- | --- | --- | --- | --- | --- | --- | --- | --- |
| Zhu AX et al[S1]. | REACH-2 | NCT02435433 | Ramucirumab vs. Placebo | Primary endpioint: Overall survival | 197 | 142 | 95 | 74 |
| Abou-Alfa GK et al [S2]. | CELESTIAL | NCT01908426 | Cabozantinib vs. Placebo | Primary endpioint: Overall survival | 470 | 317 | 237 | 167 |
| Bruix J et al [S3]. | RESORCE | NCT01774344 | Regorafenib vs. Placebo | Primary endpioint: Overall survival | 379 | 146 | 194 | 54 |
| Llovet JM et al [S4]. | BRISK-PS | NCT00825955 | Sorafenib plus Brivanib vs. Sorafenib plus Placebo | Secondary outcome: Disease-control rate | 263 | 160 | 132 | 52 |
| Zhu AX et al [S5]. | EVOLVE-1 | NCT01035229 | Sorafenib plus Everolimus vs. sorafenib plus Placebo | Secondary outcome: Disease-control rate | 362 | 203 | 184 | 83 |

Note: S means supplementary reference

Supplementary References:

S1: Zhu AX, Kang YK, Yen CJ, et al. Ramucirumab after sorafenib in patients with advanced hepatocellular carcinoma and increased alpha-fetoprotein concentrations (REACH-2): a randomised, double-blind, placebo-controlled, phase 3 trial. Lancet Oncol 2019;20:282-296.

S2: Abou-Alfa GK, Meyer T, Cheng A, et al. Cabozantinib in Patients with Advanced and Progressing Hepatocellular Carcinoma. N Engl J Med. 2018;379(1):54-63.

S3: Bruix J, Qin S, Merle P, et al. Regorafenib for patients with hepatocellular carcinoma who progressed on sorafenib treatment (RESORCE): a randomised, double-blind, placebo-controlled, phase 3 trial. Lancet 2017;389:56-66.

S4: Llovet JM, Decaens T, Raoul JL, et al. Brivanib in patients with advanced hepatocellular carcinoma who were intolerant to sorafenib or for whom sorafenib failed: results from the randomized phase III BRISK-PS study. J Clin Oncol 2013;31:3509-3516.

S5: Zhu AX, Kudo M, Assenat E, et al. Effect of everolimus on survival in advanced hepatocellular carcinoma after failure of sorafenib: the EVOLVE-1 randomized clinical trial. JAMA 2014;312:57-67.
